# Cathepsin Z/X: Breaking Down the Known and Unknown

**DOI:** 10.3390/ijms27115061

**Published:** 2026-06-03

**Authors:** Kristina Zdravkova, Milena Pavicević, Olja Mijanović, Ana Branković, Polina M. Ilicheva, Aleksandra Stankovski, Jelena Karanović, Dusan Pualić, Aleksandr A. Rubel, Ivan V. Rodionov, Lyudmila V. Savvateeva, Alessandro Parodi, Andrey A. Zamyatnin

**Affiliations:** 1 AD Alkaloid Skopje, Boulevard Alexander the Great 12, 1000 Skopje, North Macedonia; jakovlevak@hotmail.com; 2 Department of Forensic Sciences, Faculty of Forensic Sciences and Engineering, University of Criminal Investigation and Police Studies, Cara Dusana 196, 11000 Belgrade, Serbia; pavicevic.milena07@gmail.com (M.P.); ana.brankovic@kpu.edu.rs (A.B.); 3 Laboratory of Amyloid Biology, St. Petersburg State University, 199034 St. Petersburg, Russia; olja.mijanovic@gmail.com (O.M.); a.rubel@sbpu.ru (A.A.R.); 4 Institute of Chemistry, Saratov State University, 410012 Saratov, Russia; 5 Biotest AG, Landsteinerstraße 5, 63303 Dreieich, Germany; astankovski91@gmail.com; 6 Department for Human Molecular Genetics and Genomics, Institute of Molecular Genetics and Genetic Engineering, University of Belgrade, Vojvode Stepe 444A, 11042 Belgrade, Serbia; jelena.karanovic@imgge.bg.ac.rs; 7 Military Medical Academy, Crnotravska 17, 11000 Belgrade, Serbia; pualic.d@gmail.com; 8 Institute of Translational Medicine and Biotechnology, Sechenov First Moscow State Medical University, 119991 Moscow, Russia; ivan1rodionov@gmail.com (I.V.R.); ludmilaslv@yandex.ru (L.V.S.); 9 Department of Biological Chemistry, Sechenov First Moscow State Medical University, 119991 Moscow, Russia; 10 Faculty of Bioengineering and Bioinformatics, Lomonosov Moscow State University, 119234 Moscow, Russia; parodi.a@talantiuspeh.ru; 11 Scientific Center for Translation Medicine, Sirius University of Science and Technology, 354340 Sochi, Russia; 12 Belozersky Institute of Physico-Chemical Biology, Lomonosov Moscow State University, 119992 Moscow, Russia

**Keywords:** cathepsin Z/X, cysteine cathepsin, carboxypeptidase activity, integrin-binding motifs, cancer, neuroinflammation, metabolic disorders, peptidase inhibitors

## Abstract

Cathepsin Z/X (Cat Z/X) is a distinct member of the cysteine cathepsin family, known for its distinctive structural and functional properties, such as strict carboxypeptidase activity and integrin-binding motifs. These features set Cat Z/X apart from other cysteine proteases and underlie its involvement in diverse physiological and pathological processes, including immune regulation, cell signaling, and extracellular matrix remodeling. Dysregulated Cat Z/X expression and activity have been associated with various diseases, including cancer, neurodegenerative disorders, and metabolic conditions. This review summarizes current knowledge on Cat Z/X, focusing on its structural characteristics, biological functions, and roles in disease pathogenesis, particularly in malignancies, neuroinflammation, and metabolic disorders. It also explores natural and synthetic Cat Z/X inhibitors and their potential for therapeutic development. Despite growing research interest, the precise molecular mechanisms and context-specific functions of Cat Z/X are not yet fully understood. Further research is required to elucidate its regulatory networks, refine detection methods, and develop selective modulators targeting its proteolytic and non-proteolytic activities. A deeper understanding of Cat Z/X biology could pave the way for its application as a diagnostic biomarker and therapeutic target in numerous diseases.

## 1. Introduction

Cysteine cathepsins are lysosomal peptidases involved in intracellular protein turnover and various physiological and pathological processes, including antigen presentation, tissue remodeling, inflammation, and cell signaling. Beyond their traditional lysosomal functions, certain cathepsins also regulate processes at the cell surface or in the extracellular space, facilitating intercellular communication and contributing to disease progression. Extensive research has highlighted the functional diversity, regulation, and context-specific roles of cysteine cathepsins [1].

The accumulated body of research clearly shows that cathepsins play significant roles in the initiation, progression, and metastasis of tumors. Within the tumor microenvironment (TME), these enzymes participate in proteolytic remodeling, immune modulation, and signaling cascades that favor tumor survival and invasion [1].

Among them, cathepsin Z/X (Cat Z/X) has recently emerged as a peptidase of particular interest due to its distinctive structural characteristics, restricted tissue distribution, and involvement in several malignancies. As with other members of the cathepsin family, Cat Z/X undergoes a complex maturation process, progressing from pre-proenzyme forms to an active peptidase capable of executing diverse cellular functions [2]. However, despite these advances, the precise physiological and pathological roles of Cat Z/X remain insufficiently characterized. Current findings suggest that Cat Z/X may exert multifaceted effects within both intracellular and extracellular environments, potentially influencing systemic processes and tumor-associated signaling pathways. The extent to which Cat Z/X contributes to disease progression, therapeutic resistance, and patient outcomes continues to be a matter of active investigation [3].

Recent reviews have also discussed Cat Z/X [2,4,5]. Our review consolidates current knowledge on Cat Z/X, from its molecular characteristics and physiological functions to its roles in human disease and unresolved aspects of its biology. It provides an overview of Cat Z/X structure, maturation, and functions in cellular homeostasis, with particular emphasis on its emerging roles in cancer. The analysis distinguishes causal evidence from genetic and in vivo studies from purely correlative biomarker data and also summarizes current Cat Z/X inhibitors. The novelty of this work lies in connecting the structural features of Cat Z/X, including its strict carboxypeptidase activity and integrin-binding motifs, to its diverse pathophysiological roles and therapeutic potential across disease contexts.

## 2. Structure of Cathepsin Z/X

**Figure 1:**
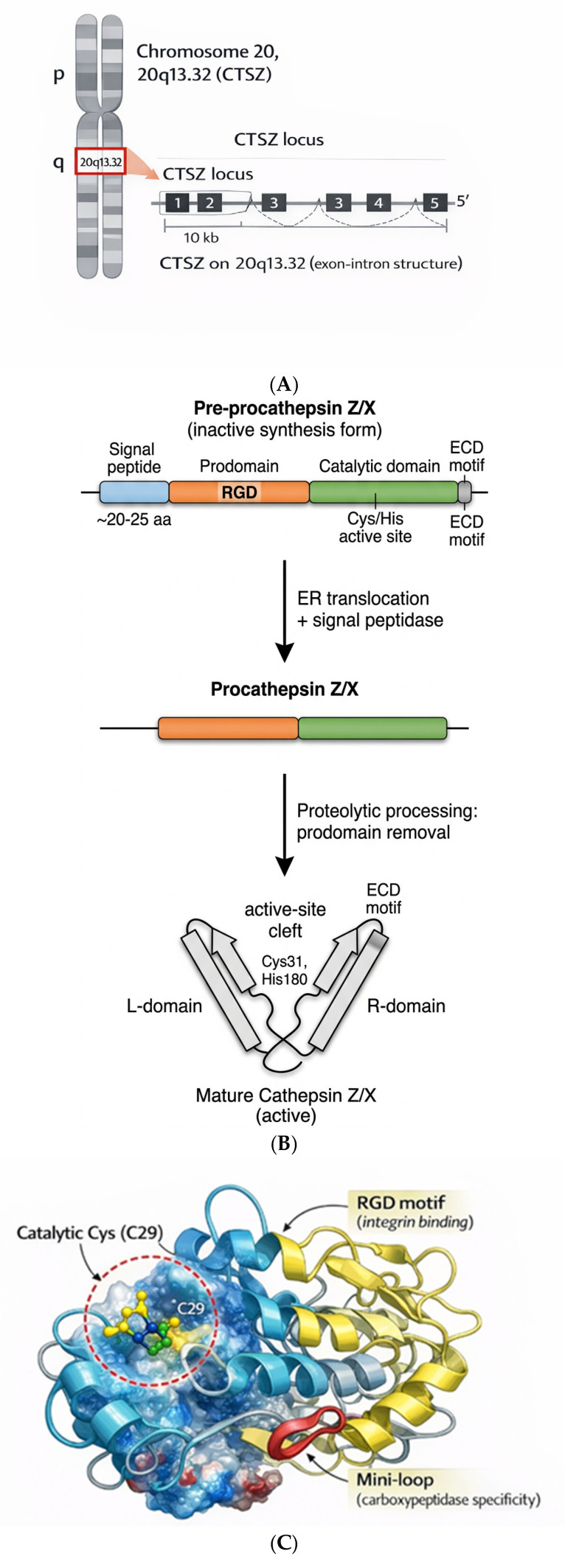
Structural organization, genomic localization, and molecular architecture of Cat Z/X: (**A**) genomic localization of the CTSZ gene on human chromosome 20 (20q13.32), showing chromosomal position and exon–intron organization within the long (q) arm; (**B**) schematic of biosynthesis and domain organization, where Cat Z/X is synthesized as an inactive precursor (procathepsin Z/X) containing an N-terminal signal peptide, a prodomain with an RGD (Arg-Gly-Asp) motif (mediating integrin-dependent, non-proteolytic signaling at the cell surface upon prodomain release during secretion), and a catalytic domain; during maturation, proteolytic removal of the prodomain exposes the catalytic cysteine residue, yielding the mature enzyme with an ECD (Glu-Cys-Asp) motif for proteolytic activity; (**C**) three-dimensional structure of mature human Cat Z/X (PDB ID: 1EF7), showing the papain-like fold, with the active-site cysteine in the catalytic cleft driving proteolysis, the mini-loop partially occluding the substrate-binding site to confer carboxypeptidase specificity, and the RGD motif mediating integrin interactions.

The *CTSZ* gene resides on chromosome 20q13 (Figure 1A), an atypical locus for human cysteine peptidases, as confirmed by in situ hybridization of PAC clones [6]. Cat Z/X was independently identified by several groups in the late 1990s, following partial sequencing of a bovine homolog in 1985 [7,8,9]. Isolation and characterization of a human brain cDNA involved comparison with the Expressed Sequence Tag (EST) database of known cysteine peptidases, revealing a 909-base-pair fragment with an open reading frame encoding 303 amino acids and a predicted molecular weight of 33.9 kDa [6]. Sequence comparisons show that Cat Z/X shares 34% identity with Cat C and 26% identity with Cat B [10]. Although it shares similarities with papain-family cysteine peptidases in its signal sequence, prodomain, and catalytic triad [11], Cat Z/X exhibits several features that distinguish it from other cathepsins.

Its structure includes three unique peptide insertions. The first, a tripeptide (His–Ile–Pro) adjacent to the glutamine residue forming the oxyanion hole, increases the distance to the catalytic cysteine, potentially influencing catalytic efficiency and substrate specificity. The others comprise an eight-residue insertion in the central region and a 14-residue insertion at the C-terminus: their functional roles remain unclear [10]. Mature Cat Z/X forms a biologically active homodimer, unlike most other monomeric lysosomal cysteine cathepsins, offering new insights into its roles in cellular processes and disease [12]. Unlike most cysteine cathepsins, which function predominantly as endopeptidases, Cat Z/X exhibits strict carboxypeptidase activity [13]. This specificity arises from a distinctive three-residue mini-loop that partially occludes the active-site cleft, restricting substrate access [14] and imposing steric constraints for carboxypeptidase reactions with negligible endopeptidase activity. This mechanism is functionally analogous to Cat B’s occluding loop [15], while the Cat Z/X mini-loop also hinders inhibition by cystatin C.

Cat Z/X is synthesized as an inactive precursor (proCat Z/X) with a short 38-residue prodomain that includes an integrin-binding Arg-Gly-Asp (RGD) motif, covalently tethered to the active site via an intramolecular disulfide bond [15]. Activation involves a regulated maturation process in which Cat L serves as a processing enzyme in vitro, though physiological mechanisms in vivo remain undefined [15]. The mature enzyme comprises 242 amino acids and contains a second integrin-binding motif, Glu-Cys-Asp (ECD) (Figure 1B,C) [15]. These motifs enable direct interactions with membrane-associated integrins and adhesion receptors, positioning Cat Z/X beyond a mere degradative peptidase [16,17]. Although *CTSZ* transcripts occur across different tissues, the protein is most abundant in immune cells (particularly macrophages, monocytes, veiled dendritic cells, bronchial epithelial cells, and alveolar type I cells), indicating roles in inflammation and immune responses [18]. It contributes to cell adhesion, bidirectional signal transduction, cell–matrix communication, and cell trafficking via binding to cell-surface heparan sulfate proteoglycans [19], with implications for tissue homeostasis and remodeling [10,20,21,22].

## 3. Physiological Functions of Cathepsin Z/X

Proteases function within intricate, interconnected networks, where modulating a single peptidase significantly impacts the activity, structure, specificity, localization, stability, and expression of others [23]. Cysteine cathepsins exemplify this through highly interconnected proteolytic networks, emphasizing their roles as signaling and regulatory enzymes rather than isolated degradative factors [5]. In a seminal study, Xu et al. [24] used chemical and genetic perturbation in dendritic cells to show that Cat Z/X deletion or inhibition alters Cat L processing and localization—including accumulation of pro- and intermediate forms alongside reduced nuclear Cat L activity. These indirect effects may reflect changes in lysosomal homeostasis (e.g., oxidative stress or pH) rather than direct proteolytic processing by Cat Z/X. Notably, these observations in defined cellular systems support a context-dependent regulatory relationship within protease networks, rather than a dominant physiological role for Cat Z/X in Cat L maturation in vivo; further studies across diverse cell types and organismal models are required [24].

Cat Z/X deficiency in murine and human fibroblasts induces accelerated cellular senescence, characterized by enlarged morphology, increased β-galactosidase activity, elevated senescence-associated markers (p16, p21, p53, caveolin), and reduced proliferative capacity. These changes accompany impaired cell migration and invasion; reintroduction of Cat Z/X reverses the senescent phenotype, confirming its essential role. Despite delayed S-phase progression in deficient cells, apoptosis does not increase, suggesting Cat Z/X promotes senescence bypass by facilitating proliferation and motility [25].

Cat Z/X critically regulates T-cell migration by modulating LFA-1 integrin activity [26]. Its overexpression increases T-cell motility on ICAM-1-expressing cells (e.g., endothelial cells) while reducing stable leukocyte adhesion. Mechanistically, Cat Z/X cleaves the β2 cytoplasmic tail of LFA-1 at C-terminal residues controlling α-actinin-1 binding—a cytoskeletal linker governing LFA-1 lateral mobility. Pharmacological inhibition or silencing reinforces LFA-1–ICAM-1 interactions, promoting adhesion and impairing migration. In activated T cells, Cat Z/X translocates to the plasma membrane, co-localizing and interacting with LFA-1 to post-translationally tune integrin function, influencing motility, proliferation, and immune activation [26,27]. The proenzyme of Cat Z/X also promotes T-cell adhesion, phagocytosis, and activation [22].

During dendritic cell (DC) maturation, extensive morphological remodeling occurs under multiple signaling pathways. Cat Z/X contributes by regulating podosome formation; upon plasma membrane translocation, it activates the integrin receptor Mac-1 (CD11b/CD18), facilitating substrate binding for full maturation [28]. Xu et al. showed Cat Z/X expression increases in immortalized murine Mutu DCs following TLR9 activation via IL-6 secretion [28]. Obermajer et al. [22] demonstrated that cysteine cathepsin inhibitors E-64 and CA-074, plus monoclonal antibody 2F12, attenuate U-937 cell adhesion to polystyrene- and fibrinogen-coated surfaces by inhibiting Mac-1. Procathepsin Z/X colocalizes with β2 and β3 integrin subunits, while the mature enzyme preferentially associates with β2 integrins [22].

Emerging evidence links Cat Z/X to NLRP3 inflammasome activation in macrophages: notably, secreted Cat Z/X binds α5β1 integrin on the cell surface, triggering caspase-1 processing and IL-1β production independently of its enzymatic activity. This non-proteolytic extracellular signaling enhances inflammasome responses to stimuli like silica crystals without altering pyroptosis or inflammasome gene expression, complementing Cat Z/X’s traditional proteolytic roles and underscoring its duality in inflammation [29,30]. These properties make Cat Z/X a promising target for selective inhibitors to elucidate its biological roles.

## 4. Cathepsin Z/X as a Diagnostic and Therapeutic Target in Cancer, Inflammation, and Other Diseases

### 4.1. Epigenetic Activation and Non-Proteolytic Cat Z/X Signaling in Cancer

Cat Z/X is a promising diagnostic and therapeutic target in cancer and inflammation. Advances in detection methods now allow in vivo monitoring of its precursor and mature forms [2,3]. Traditional detection methods (e.g., internally quenched fluorogenic substrates) often perform poorly in complex biological samples like cell lysates and plasma. To address this, Nägler et al. developed a highly specific and sensitive ELISA for quantifying human (pro)Cat Z/X in plasma and serum, enhancing its utility as a diagnostic marker [3]. Activity-based probes (ABPs) targeting active Cat Z/X—such as Cy5-DCG04, GB123, MGP140, and MGP302 (dimethyl sulfoxonium ylide electrophiles)—also show promise, though their sensitivity and specificity require further refinement [31,32]. The functional role of Cat Z/X remains somewhat elusive, primarily due to variable localization, different maturation states, and interactions with endogenous inhibitors.

Biotinylated suicide inhibitors combined with laser scanning confocal microscopy enable precise spatial and temporal visualization of Cat Z/X activity in living tissues. These inhibitors covalently bind the active site, irreversibly labeling only enzymatically active peptidases; when conjugated to tags such as biotin, they allow high-resolution imaging at cellular and subcellular levels in animal models. Gounaris et al. used this approach to visualize cysteine cathepsin activity during intestinal tumorigenesis in mice, revealing uneven distribution of Cat Z/X with enrichment in areas of focal inflammation, angiogenesis, and polyp growth [33]. Elevated activity of Cath B and Cat Z/X in these tissues suggests involvement in ECM remodeling, immune cell infiltration, and neovascularization—hallmarks of tumor progression. Schwenck et al. applied similar methods to study delayed-type hypersensitivity, demonstrating that cysteine cathepsins, including Cat Z/X, regulate the effector phase of inflammation through immune activation and tissue remodeling, thereby linking these proteases to tumorigenesis [33,34].

Epigenetic regulation, particularly DNA hypomethylation of the *CTSZ* promoter, drives Cat Z/X overexpression in cancer. In clear cell renal cell carcinoma (ccRCC), *CTSZ* hypomethylation correlates with poor chemotherapy response, higher mortality, and aggressive tumor behavior. Zhang et al. linked macrophage-derived cathepsins, including Cat Z/X, to ccRCC prognosis and tumor microenvironment composition; elevated Cat Z/X correlates with increased immune infiltration, enhanced tumor-associated macrophage activity, M2 polarization, pro-tumorigenic signaling, and immunosuppressive microenvironments that promote tumor progression, immune evasion, and therapy resistance [35].

Research on Cat Z/X can be broadly divided into two categories. Functional evidence (from cellular and in vivo models) shows that genetic deletion, knockdown, or targeted manipulation of Cat Z/X directly influences disease-relevant processes. Correlative data describe associations between Cat Z/X expression levels and pathological states, suggesting diagnostic/prognostic potential but not direct mechanistic involvement. Distinguishing between these approaches is essential to avoid overestimating Cat Z/X’s causal role in disease pathogenesis [21,29,36].

Cat Z/X is expressed in many human cancer cell lines and primary tumors, where increased levels associate with advanced disease stages and greater tumor aggressiveness [6,37]. In the PyMT mammary carcinoma model, Sevenich et al. showed that Cat Z/X deficiency leads to compensatory upregulation of Cat B, indicating synergistic relationships between these cathepsins [38]. Subsequent work highlights non-proteolytic functions: Akkari et al. reported that Cat Z/X promotes tumor cell invasion via integrin-mediated signaling rather than catalytic activity [39].

The prodomain of Cat Z/X contains an RGD motif removed upon enzymatic activation [15]. This motif mediates cell-surface binding and influences cell migration and adhesion. Cat Z/X has been implicated in epithelial–mesenchymal transition (EMT) and interacts with integrins including αvβ3 in immune-related processes such as dendritic cell maturation and lymphocyte activity [40]. In cancer cells, tumor-promoting effects are primarily mediated by the RGD-containing prodomain rather than proteolytic activity; deletion or mutation of this motif disrupts FAK–Src signaling and focal adhesion complex formation, impairing cell proliferation [39].

Kos et al. concluded that, contrary to expectations, Cat Z/X is not primarily involved in ECM degradation during tumor invasion and metastasis; instead, it plays roles in phagocytosis and immune regulation due to its restricted expression in immune cells [18]. Subsequent studies show that Cat Z/X regulates clathrin-mediated endocytosis through proteolytic processing of profilin-1, thereby influencing tumor cell migration and invasion [41].

Context-dependent functions are evident in immune cells. While Cat Z/X colocalizes with LFA-1 in T cells, it does not colocalize at the plasma membrane in NK-92 cells used for adoptive cellular immunotherapy [41]. Inhibition of Cat Z/X does not affect NK-92 immunoconjugate formation with target cells (an LFA-1-dependent process); instead, Cat Z/X redistributes to cytotoxic granules and is secreted during degranulation, indicating context-dependent roles in immune-mediated antitumor responses [42].

An additional layer of complexity involves peptidase expression and regulation within tumor-associated stromal cells (endothelial cells, fibroblasts, immune cells, mesenchymal stem cells) [43]. Stromal peptidase regulation often deviates from that in cancer cells, leading to context-dependent effects on tumor progression [44]. In the Rip1-Tag2 PanNET mouse model, Cat B and Cat S produced by tumor-associated macrophages were identified as primary catalysts of tumor growth, angiogenesis, and invasion in vivo [45]. These findings indicate that peptidases from stromal versus cancer cells can have differing, sometimes opposing, effects depending on the cancer type. Moreover, tumor heterogeneity and adaptive evolution shape peptidase networks within the tumor microenvironment, as selective pressures from cell–cell interactions can induce compensatory upregulation of other peptidases in response to loss or inhibition of specific peptidases [46].

### 4.2. Cancer Types Associated with Cat Z/X

Cat Z/X differs from other cathepsins, such as B, C, and S, in its roles in tumor and immune processes. As summarized in Table 1, Cat Z/X has been linked to both proteolytic and non-proteolytic functions in cancer progression and inflammation, whereas the other cathepsins are more defined roles in invasion, antigen presentation, and immune regulation [2,4,47]. Table 1 provides a concise overview of the main functional distinctions and disease associations discussed in this section. It should be noted that the available evidence for Cat Z/X is context dependent across cancer types. In breast cancer, reduced Cat Z/X expression has been associated with a potentially protective phenotype, whereas in gastric, colorectal, and lung cancers the evidence more consistently supports tumor-promoting roles [48]. Together, these findings suggest that the biological impact of Cat Z/X may vary according to tumor type, cellular context, and disease stage.

*Gastric cancer.* Cat Z/X is markedly upregulated during *Helicobacter pylori* infection and associated gastritis [49,50]. In patients who do not respond to antibiotic therapy, Cat Z/X levels are elevated. Cat Z/X may promote tumor progression by enhancing β_2_ integrin (Mac-1)-dependent immune activation, increasing MHC class II expression, and driving dysregulated cytokine signaling characterized by imbalanced IFN-γ and IL-4 production [46]. In addition Cat Z/X expression in monocytes is induced by soluble mediators from *H. pylori*-infected epithelial cells and correlates with increased cancer cell invasiveness [50]. Both macrophages and gastric epithelial cells upregulate Cat Z/X through CagA-dependent, cytokine-driven pathways involving ERK1/2 signalling in macrophages and JNK signalling in epithelial cells [51]. Cat Z/X knockdown induces G1 cell-cycle arrest and apoptosis, whereas interaction with ribosomal protein P0 confers anti-apoptotic effects [52].

*Colorectal cancer.* Fang et al. demonstrated that the histone methyltransferase KMT2A is overexpressed in colorectal cancer and promotes Cat Z/X transcription via H3K4 trimethylation of the Cat Z/X promoter, requiring p65-mediated KMT2A recruitment [53]. Serum Cat Z/X levels does not reliably distinguish colorectal cancer from healthy individuals or benign lesions [54], but higher serum levels correlate with shorter overall survival in stage I–III patients who did not receive chemotherapy [55]. Jechorek et al. found that Cat Z/X is associated with early tumorigenesis, with highest epithelial expression in high-grade intraepithelial neoplasia and early-stage carcinoma compared with adenoma and normal mucosa [56]. Loss of Cat Z/X expression is linked to tumor cell detachment, invasion, disease progression, and poorer survival, whereas expression in tumor-associated macrophages may reflect an antitumor immune response [56].

*Breast cancer.* Significantly lower levels of Cat Z/X and Cat H have been reported in inflammatory breast cancer [57]. Deficiencies in both Cat Z/X and Cat B produces stronger anticancer effects than loss of either enzyme alone, suggesting partial functional compensation [58]. Hypomethylation of the *CTSZ* gene in peripheral blood from young Chinese women may serve as a biomarker for early-stage breast cancer [57]. Additional studies suggest that Cat Z/X may be associated with a protective phenotype in in situ breast cancer through modulation of oncogenic drivers such as, PELATON, cryptochrome 2 (*CRY2*), and peroxiredoxins, and that coordinated changes with Cat F influence breast cancer risk and progression [48].

*Pancreatic cancer.* Silencing Cat Z/X significantly reduces pancreatic cancer cell adhesion by disrupting interactions with S100P-binding protein and RGD-binding integrins, particularly αvβ5 [59].

Hepatocellular carcinoma. Cat Z/X overexpression promotes metastasis by inducing epithelial–mesenchymal transition (EMT), with increased fibronectin and vimentin and reduced E-cadherin and α-catenin [60]. Quantitative proteomic analyses also show Cat Z/X upregulation in gallbladder cancer [61].

*Skin cancer.* Elevated Cat Z/X expression, together with osteonectin/SPARC and macrophage migration inhibitory factor, modulates melanoma cell-cycle progression and angiogenesis [62]. Co-culture studies with adipose-derived and bone marrow stromal cells show that increased Cat Z/X enhanced angiogenesis [74].

*Lung cancer.* Serum Cat Z/X and cystatin C are significantly elevated in lung cancer patients and correlate with adverse clinicopathological features, reduced overall survival, and poorer prognosis. Deguelin suppresses non-small cell lung cancer metastasis by inhibiting Cat Z/X–FAK signaling [63,64].

*Prostate cancer.* Cat Z/X protein levels rise in prostatic intraepithelial neoplasia and prostate carcinoma without matching mRNA changes, suggesting post-transcriptional regulation [65]. Reduced *Cat Z/X* mRNA in peripheral immune cells serves as a diagnostic/prognostic biomarker [66]. High Cat Z/X expression activates IGF/FAK signaling and correlates with advanced stage, higher Gleason score, increased tumor mutation burden, and poorer outcomes [67]. Cat Z/X suppression impairs proliferation, migration, invasion, and colony formation [68,69]. Mechanistically, Cat Z/X cleaves the C-terminal tyrosine of profilin 1, disrupting clathrin-mediated endocytosis and promoting motility; these effects are reversed by Cat Z/X inhibition or non-cleavable profilin 1 mutants [69].

*Glioblastoma*. Elevated *Cat Z/X* in glioblastoma correlates with reduced survival and poorer functional status [70,71]. Expression peaks in the mesenchymal subtype and shows heterogeneous distribution across tumor regions [72]. Cat Z/X is also present in CD133-positive stem cell niches around SDF-1α/CD68-expressing arterioles, suggesting a role in stem cell homing and niche regulation [72].

*Kidney cancer.* Comparative analyses of embryonic kidney cells and renal carcinoma lines show higher *Cat Z/X* expression and nuclear localization in tumor cells, with mature forms indicating enhanced proteolytic activity associated with malignant transformation [34,73,75]. High *Cat Z/X* also occurs in invasive somatotroph pituitary neuroendocrine tumors, where it promotes growth and invasion [76,77].

Accumulating evidence indicates that Cat Z/X, predominantly derived from immune cells, contributes to cancer progression through both proteolytic and non-proteolytic mechanisms. Its secretion may facilitate communication within the tumor microenvironment and has been linked to invasion, tumor progression, and cell-death regulation. These findings suggest that effective therapeutic strategies should target both catalytic activity and non-proteolytic, integrin-mediated functions.

### 4.3. Neurodegeneration and Neuroinflammation

**Figure 2:**
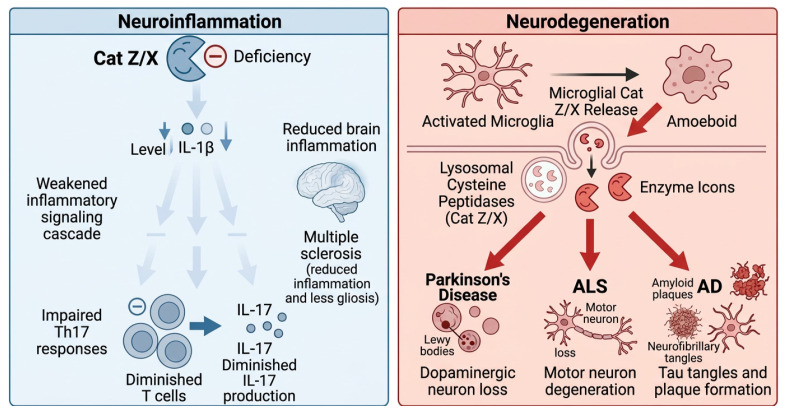
Schematic diagram illustrating the dual roles of Cat Z/X in neurodegeneration and neuroinflammation. Note: Parkinson’s disease and ALS feature intertwined neuroinflammation and neurodegeneration, and Cat Z/X contributes to both processes.

Neurodegenerative proteinopathies—including Alzheimer’s disease, Parkinson’s disease, frontotemporal dementia, Huntington’s disease, and ALS—feature toxic protein aggregates that activate microglia and trigger chronic neuroinflammatory cascades [78]. Activated microglia release lysosomal cysteine peptidases, including Cat Z/X, alongside IL-1β and TNF-α; concurrent endolysosomal dysfunction promotes accumulation of neurotoxic proteins and undegraded cathepsin substrates, amplifying neuronal injury (Figure 2) [4,79]. Conversely, Cat Z/X deficiency is associated with multiple sclerosis (MS), where reduced IL-1β production attenuates inflammatory signaling and Th17 responses—a key ameliorative mechanism [80].

Cat Z/X, highly expressed in immune and neural cells, links inflammation to neurodegeneration by modulating β2 integrin-dependent immune responses and by cleaving C-terminal dipeptides from α- and γ-enolase, thereby abolishing their neurotrophic support for neuronal survival and neurite outgrowth [36,81]. In oligodendroglial cells, Cat Z/X negatively regulates differentiation: its inhibition accelerates maturation to myelinating γ-enolase-active oligodendrocytes and enhances remyelination [4,36]. Cat Z/X-mediated truncation of γ-enolase also prevents its γ1-syntrophin-dependent plasma membrane translocation, dampening neurotrophic signaling, neuritogenesis, and neuronal survival; similar enolase alterations contribute to metabolic reprogramming and migration in cancer cells [81].

Genetic and experimental models further implicate Cat Z/X in specific diseases. In MS, Cat Z/X deficiency reduces IL-1β production, attenuates Th17 responses, and ameliorates inflammation [36]. In CLN7 neuronal ceroid lipofuscinosis, disturbed Cat Z/X synthesis and activity—together with SCMAS and saposin D accumulation—impair autophagy, leading to lipofuscin-like storage and ATP synthase subunit c buildup.

PD is a neurodegenerative disorder characterized by progressive loss of dopaminergic neurons in the substantia nigra pars compacta (SNc). Its pathogenesis involves glial activation, T-cell proliferation, and elevated pro-inflammatory cytokines; lysosomal peptidases such as Cat Z/X contribute to neuroinflammatory responses and neurotoxicity. In a 6-OHDA rat model of PD (unilateral medial forebrain bundle lesioning), Cat Z/X expression, protein levels, and enzymatic activity increase in the ipsilateral SNc. At early stages, it localizes primarily to neuronal lysosomes, coinciding with rapid loss of tyrosine hydroxylase (TH)-positive neurons. Within 12 h of 6-OHDA injection, Cat Z/X associates with microglial cells; after 4 weeks, it predominates in activated microglia concentrated in the SNc. These shifts confirm Cat Z/X as a proteolytic mediator of dopaminergic neurodegeneration [82].

Transcriptomic analyses of ALS mouse models reveal altered expression of lysosomal and proteolytic genes, including cathepsins, suggesting their involvement in disease pathology and utility as diagnostic biomarkers and therapeutic targets. ALS features progressive degeneration of upper and lower motor neurons, causing muscle weakness, paralysis, and impaired speech, swallowing, and respiration [83].

AD involves early neuroinflammation, Aβ plaques, microglial activation, and cytokine release (TNF-α, IL-1β), with Cat Z/X upregulated in microglia associating with plaques—supporting biomarker/therapeutic potential [84]. In a conditional PS1/PS2 double knockout mouse model, presenilin loss induces gliosis and elevates cathepsin S, Cat Z/X, chemokines (Ccl3/Ccl4), and complement (C1qb/C3/C4), triggering protease/immune neuroinflammation [85]. Long-term urolithin A—a mitophagy promoter—improves lysosomal function and normalizes Cat Z/X in APP/PS1 mice, highlighting therapeutic promise [86].

In Huntington’s disease and related polyglutamine disorders, cathepsin L and Cat Z/X contribute to proteolytic processing of polyglutamine-rich aggregates [87]; Cat Z/X, together with bleomycin hydrolase, has been proposed as both a disease biomarker and therapeutic target [88].

Diseases associated with Cat Z/X and their pathological features are summarized in Table 2.

### 4.4. Other Diseases Associated with Cat Z/X

*Respiratory silicosis* arises from the inhalation of crystalline silica particles into the lungs, causing chronic inflammation and impaired pulmonary function. Silica deposition activates alveolar macrophages, triggering inflammatory signaling pathways, including NLRP3 inflammasome activation and subsequent IL-1β secretion. Experimental studies demonstrate that extracellular Cat Z/X plays a critical role in silica-induced NLRP3 inflammasome activation and IL-1β production. Notably, while Cat Z/X−/− THP-1 macrophages induce an NLRP3-dependent response, NLRP3−/− THP-1 cells fail to secrete IL-1β despite Cat Z/X release. Co-culture of Cat Z/X−/− and NLRP3−/− THP-1 cells partially restores IL-1β production, underscoring Cat Z/X’s distinct contribution to inflammasome activation, likely mediated by its integrin-binding domain. Genetic ablation studies further confirm Cat Z/X’s pathological role: deficient mice show impaired inflammatory responses, including reduced IL-1β production and altered macrophage activation, indicating a specific contribution to immune-mediated pathology rather than redundant housekeeping activity [29].

*Primary biliary cholangitis (PBC)* is a chronic autoimmune liver disease characterized by progressive destruction of intrahepatic bile ducts, leading to cholestasis, cirrhosis, jaundice, and ultimately end-stage liver failure. Three clinical forms of PBC are recognized: a slowly progressive form with favorable survival; a progressive form that advances to cirrhosis or portal hypertension; and an advanced form that culminates in jaundice and hepatic failure. Genetic studies in the Japanese population have identified polymorphisms (rs13720 and rs163800) within the *NELFCD/CAT Z/X* locus that are associated with more severe disease progression [89]. Recent evidence indicates that Cat Z/X levels increase with advancing disease stage and correlate significantly with hepatic biochemical markers, including alanine aminotransferase, aspartate aminotransferase, platelet count, total bilirubin, and albumin. In late-stage PBC, Cat Z/X shows significant upregulation in hepatocytes and redistributes from the peribile canalicular membrane to the cytoplasm, accompanied by a loss of colocalization with the lysosomal marker LAMP1. Collectively, these observations suggest that dysregulated expression and subcellular localization of Cat Z/X contribute to cholestatic injury and disease progression in PBC [42].

*Osteoporosis* arises from an imbalance between bone-resorbing osteoclasts of monocytic origin and bone-forming osteoblasts derived from mesenchymal stem cells. Results showed that Cat Z/X levels were significantly higher in peripheral blood mononuclear cells (PBMCs) from women over 50 years of age with osteopenia or osteoporosis compared with age-matched controls without bone loss. These findings suggest that Cat Z/X may serve as a potential biomarker for the early detection of osteoporosis [90]. Proteases are also critical regulators of hematopoietic stem and progenitor cell (HSPC) mobilization from bone marrow niches [91].

*Pulmonary arterial hypertension (PAH)* is a chronic, progressive disorder characterized by elevated pulmonary arterial pressure, leading to right-sided heart failure and premature mortality. Epigenetic analyses have identified hypermethylation at three loci in PAH, including *CTSZ* (cg04917472), conserved oligomeric Golgi complex 6 *COG6* (cg27396197), and zinc finger protein 678 *ZNF678* (cg03144189) [92]. Hypermethylation of the *CTSZ* locus correlates with reduced *CTSZ* mRNA expression in peripheral blood mononuclear cells and is associated with increased caspase-3/7 activity and enhanced apoptosis. Reduced Cat Z/X levels may therefore contribute to endothelial cell loss and pulmonary vascular remodeling. These findings suggest that epigenetic silencing of *CTSZ* represents a molecular link between DNA methylation, apoptotic signaling, and vascular injury in PAH, positioning Cat Z/X as both a biomarker and potential pathogenic factor [92].

*Obstructive sleep apnea (OSA)* is a prevalent sleep-related breathing disorder characterized by recurrent upper airway obstruction and intermittent hypoxia. Data-independent acquisition quantitative proteomics identified multiple serum and urinary proteins with differential expression in children with OSA compared to healthy controls. Among these, Cat Z/X showed a positive correlation with disease severity, whereas sex hormone-binding globulin (SHBG) showed a negative correlation. Independent validation in an external cohort using ELISA confirmed strong diagnostic performance, with area under the curve (AUC) values of 0.863 for Cat Z/X and 0.738 for SHBG. These findings highlight the potential of Cat Z/X as a multifaceted biomarker for diagnosis and severity assessment in pediatric OSA [93].

As shown in Table 2, Cat Z/X contributes to multiple diseases via distinct mechanisms.

## 5. Inhibitors of Cat Z/X

Paulick & Bogyo and Sadaghiani et al. demonstrated that selective inhibition of Cat Z/X requires careful consideration of inhibitor specificity, as well as experimental conditions that minimize off-target effects on other lysosomal proteases [31,94].

It is important to note that a substantial body of evidence suggests that many physiological and pathological functions of Cat Z/X are independent of its catalytic activity. Instead, these functions rely on non-catalytic mechanisms, including integrin-mediated interactions at the cell surface [29,95,96]. Consequently, inhibition of enzymatic activity alone may not fully recapitulate the effects of genetic deletion or disruption of integrin-binding functions, underscoring the need to distinguish between catalytic and non-catalytic roles when interpreting inhibitor studies [5].

To date, no highly specific endogenous inhibitors of Cat Z/X have been identified, although some cystatins exhibit weak or context-dependent inhibition [96,97]. This reduced sensitivity to cystatins is consistent with the structural features of Cat Z/X. By comparison, monoclonal antibody 2F12, which targets the mature enzyme, has been shown to effectively inhibit Cat Z/X activity [18]. Additionally, Klemencic et al. reported inhibition of Cat Z/X by synthetic Cat B inhibitors CA-074 and GFG-semicarbazone [97], further demonstrating the potential for exogenous modulation [97]. In contrast to exopeptidases such as Cat B and Cat C, Cat Z/X exhibits reduced sensitivity to inhibition by the p41 fragment (thyropins) [98]. The partial resistance of Cat Z/X to cystatins and thyropins is explained by steric hindrance caused by the characteristic "mini-loop" residues projecting into the active-site cleft of the homodimer [12].

These findings confirm that endogenous protein inhibitors play no significant role in regulating Cat Z/X activity, distinguishing it from other cysteine cathepsins. This underscores the need for synthetic approaches to modulate its activity.

The first synthetic small-molecule irreversible inhibitor reported as having preferential selectivity for Cat Z/X is AMS 36, an epoxysuccinyl-based peptidomimetic with nanomolar potency [94]. Irreversible inhibitors act by covalent modification of the catalytic cysteine, providing potent and sustained target engagement, but they often show only moderate-to-low selectivity across papain-family proteases and carry a risk of off-target cysteine alkylation that can complicate interpretation of in vivo phenotypes. Cellular permeability of these compounds is variable and frequently limited by polar electrophilic warheads and peptidic backbones. Although long target residence time can be advantageous, the intrinsic reactivity and associated pharmacokinetic and safety liabilities complicate dosing and use [99]. AMS 36 demonstrates preferential activity against Cat Z/X but exhibits some cross-reactivity with Cat B in certain tissues [94]. Another E 64-derived compound, nPrNH-(2S,3S)-tEps-Ile-OH, has modest potency yet retains approximately tenfold selectivity over Cat B and Cat L [14].This pattern may reflect differences in reactivity: while the parent compound **E64** shows weak inhibition of Cat Z/X, its derivatives (such as AMS 36) achieve higher potency through structural optimization [14,97]. Importantly, phenotypes observed in Cat Z/X-deficient models closely mirror those produced by pharmacological inhibition, suggesting on-target effects and supporting the therapeutic potential of selective Cat Z/X inhibitors in pathological inflammation, tumor migration, and neurodegeneration [100].

Reversible, selective triazole-based inhibitors of Cat Z/X have been described, with compound **Z9** (also termed compound **22**) exhibiting the lowest inhibition constant and good selectivity over Cat B, Cat L, Cat H, and Cat S [100]. These agents act via non-covalent binding to active-site pockets and subpockets, which enables finer selectivity tuning through structure–activity relationship studies and results in lower reactivity-related toxicity compared to covalent inhibitors [100,101]. Importantly, reversible scaffolds often demonstrate improved cellular permeability—they can be smaller and less polar—making them more amenable to optimization for robust cellular activity. Structure–activity relationship analyses further reveal that the central ketomethylenethio linker between the benzodioxine and triazole moieties is essential for potency, whereas substitutions on the triazole ring have limited impact [101]. In vivo, these reversible inhibitors are expected to offer improved safety and dose control. However, pharmacokinetic optimization remains necessary to ensure adequate exposure and target engagement, typically requiring medicinal chemistry efforts to enhance pharmacokinetics and cellular potency to match the efficacy of irreversible inhibitors [101].

Several Cat Z/X inhibitors have been evaluated beyond purified-enzyme assays and show clear functional effects in vivo. AMS-36 exerts protective effects against LPS-induced striatal degeneration in rodent models, consistent with modulation of neuroinflammatory pathways [102]. In breast cancer models (FVB/PyMT and MMTV-PyMT), chronic administration of the reversible inhibitor Z9 significantly impaired tumor progression, growth, and metastasis by reducing tumor cell migration and invasion [101]. In contrast, several newer triazole- and benzodioxine-derived inhibitors remain characterized primarily at the enzymatic level: they display reported selectivity and in vitro potency but lack demonstrated in vivo functional outcomes such as modulation of inflammation, tumor migration, or neuronal survival [100]. For mechanistic interpretation, it is therefore important to distinguish tools validated in vivo (e.g., AMS-36, Z9) from those restricted to biochemical and cell-based characterization [101,103].

Despite these advances, selective Cat Z/X inhibitors remain limited. Targeting Cat Z/X holds context-specific therapeutic promise for inflammatory, oncological, and neurodegenerative conditions, underscored by its lack of endogenous regulation—a feature that may enhance its targetability in specific pathological contexts [102]. An overview of key synthetic inhibitors is provided in Table 3.

Taken together, these findings indicate that therapeutic targeting of Cat Z/X is complex and highly context-dependent, varying by tissue type, disease stage, and cellular microenvironment. As many of its biological functions are mediated through non-proteolytic, integrin-dependent mechanisms, inhibiting catalytic activity alone is unlikely to fully recapitulate the effects observed in genetic deletion models. Therefore, future strategies should explicitly distinguish between its enzymatic and scaffolding functions—for example, by developing tools to selectively modulate each—and ensure rigorous validation in physiologically relevant disease models.

## 6. Conclusions

Cat Z/X is a distinctive cysteine peptidase with strict carboxypeptidase activity, integrin-binding motifs, and broad roles in diverse physiological and pathological processes. Knockout and inhibition studies underscore its therapeutic potential, and its dysregulation in cancer, neurodegenerative disorders, and metabolic disease positions it as both a biomarker and a drug target, highlighting its context-dependent nature and the need to consider both catalytic and non-catalytic functions. Although advances have clarified its structure and function, key gaps persist in understanding its molecular mechanisms, context-specific pathology, and non-catalytic signaling. To address these gaps, future work should adopt an integrated strategy: (i) Combine cell-type-resolved genetics (conditional knockouts/knock-ins, lineage tracing) with spatial and functional readouts (single-cell and spatial omics, activity-based probes) to disentangle immune vs. tumor cell effects in cancer and distinguish catalytic vs. non-catalytic roles; (ii) Develop advanced diagnostics that assess: (1) total abundance (immunoassays), (2) active fraction (activity-based probes), and (3) anatomical source (spatial profiling combined with plasma/serum measurements) to distinguish pro-forms from the active enzyme and local tissue activity from systemic levels; (iii) Design selective inhibitors that exploit unique structural features, such as the active-site “mini-loop,” to minimize off-target effects.

Prioritizing these efforts will sharpen mechanistic understanding and accelerate translation into clinical applications, especially in heterogeneous diseases like cancer where precise spatial and functional resolution is critical.

## Figures and Tables

**Table 1:** Malignancies associated with Cat Z/X.

Cancer Type	Role/Mechanism	Key Findings	Evidence Level	Refs.
Gastric	Promotes progression via immune activation, cytokine dysregulation	Upregulated in *H. pylori* infection/cancer; knockdown induces G1 arrest/apoptosis	Both functional studies and clinical correlations	[49,50,51,52]
Colorectal	Transcriptional regulation; dual role (epithelial pro-tumor, macrophage anti-tumor)	Promoter methylation via KMT2A; high serum → poor survival; loss → invasion	Primarily correlative, with limited mechanistic support	[53,54,55,56]
Breast (inflammatory)	Protective in situ; biomarker via hypomethylation	Low Cat Z/X + Cat H/B → anticancer synergy	Largely correlative, with additional support from deficiency and biomarker studies	[48,57,58]
Pancreatic	Tumor cell adhesion via integrins (αᵥβ_5_)	Silencing reduces adhesion	Mainly based on in vitro functional studies	[59]
Hepatocellular	EMT/metastasis promoter	↑ Mesenchymal markers, ↓ epithelial; also ↑ in gallbladder	Functional evidence supported by proteomic data	[60,61]
Skin (melanoma)	Angiogenesis, cell-cycle modulation	With SPARC/MIF; stromal co-cultures enhance angiogenesis	Mainly on correlative expression data and in vitro co-culture models	[62]
Lung	Biomarker; FAK signaling	↑ Serum → poor prognosis; deguelin inhibits metastasis	Mixed correlative clinical data with functional findings from experimental metastasis models	[63,64]
Prostate	Proliferation/invasion via profilin 1 cleavage, FAK/IGF	Post-transcriptional ↑; suppression blocks motility/endocytosis	Functional data with clinical correlations	[65,66,67,68,69]
Glioblastoma (mesenchymal)	Progression, stem cell niche/homing	↑ With Cat K; heterogeneous expression	Primarily based on patient-derived and in silico data	[70,71,72]
Kidney (renal carcinoma)	Nuclear localization, proteolytic activity	Mature forms ↑ in transformation; also in pituitary NETs	Comparative expression studies and functional insights from tumor cell lines	[73]

Abbreviations: ↑, increased/upregulated; ↓, decreased/downregulated; →, associated with or indicating direction of effect.

**Table 2:** Diseases Associated with Cat Z/X and Their Pathological Features.

Disease	Pathological Features	Role of Cat Z/X	Refs.
Neurodegenerative disorders (general)	Proteinopathies (e.g., AD, PD, etc.) with microglial activation, lysosomal dysfunction, neurotoxic cascades	Released by activated microglia; cleaves enolase (impairs neurotrophic activity, neuritogenesis); inhibits oligodendrocyte differentiation	[4,79,81]
Alzheimer’s disease (AD)	Early neuroinflammation, Aβ plaques, synaptic/neuronal loss, gliosis; PS1/PS2 mutations in familial cases	Upregulated in microglia, associates with plaques; elevated in gliosis models; normalized by urolithin A	[84,85,86]
Parkinson’s disease (PD)	Progressive loss of dopaminergic neurons in substantia nigra; glial activation, T-cell proliferation, pro-inflammatory cytokines	Elevated mRNA/protein/activity in SNc; localizes to neurons then microglia; proteolytic mediator	[82]
Frontotemporal dementia	Proteinopathies with misfolded protein accumulation.	Released by microglia contributing to neuronal damage	[78]
Huntington’s disease	Polyglutamine protein misfolding and aggregation	Cleaves polyglutamine aggregates; proposed biomarker/target	[87,88]
Amyotrophic lateral sclerosis (ALS)	Progressive degeneration of upper and lower motor neurons, leading to muscle weakness, paralysis, and impaired functions.	Altered expression in transcriptomic analyses; potential biomarker/target	[83]
Multiple sclerosis (MS)	Diminished IL-1β production attenuates inflammatory signaling and Th17 responses	Deficiency ameliorates disease by reducing IL-1β and Th17 responses	[80]
Neuronal ceroid lipofuscinosis type 7 (CLN7)	Lysosomal dysfunction, accumulation of autofluorescent lipofuscin-like pigments, subunit c of ATP synthase, and impaired autophagy	Elevated expression with lysosomal dysfunction and impaired autophagy	[36]
Respiratory silicosis	Inhalation of silica particles causes chronic lung inflammation, NLRP3 inflammasome activation, IL-1β secretion, and impaired pulmonary function	Extracellular Cat Z/X drives NLRP3 activation via integrin-binding domain; elevated in BALF; deficiency reduces IL-1β	[29]
Primary biliary cholangitis (PBC)	Autoimmune destruction of intrahepatic bile ducts leads to cholestasis, cirrhosis, jaundice, and liver failure; three clinical forms (slow, progressive, advanced)	Genetic polymorphisms (rs13720, rs163800) linked to severe progression; upregulated in late stages, correlates with liver markers	[42,89]
Osteoporosis	Imbalance of osteoclast bone resorption and osteoblast formation; elevated in postmenopausal women with osteopenia/osteoporosis	Higher in PBMCs (early biomarker); pro-Cat Z/X reduces HSPC adhesion, regulates SDF-1	[90,91]
Pulmonary arterial hypertension (PAH)	Elevated pulmonary pressure causes right heart failure; involves vascular remodeling and endothelial apoptosis	*CTSZ* hypermethylation reduces expression, links to apoptosis; biomarker/pathogenic	[92]
Obstructive sleep apnea (OSA)	Recurrent airway obstruction and hypoxia, especially in children; correlates with disease severity	Elevated serum/urinary levels correlate with severity; diagnostic biomarker (AUC 0.863)	[93]

**Table 3:** Synthetic Inhibitors of Cat Z/X.

Inhibitor	Type	Structure/Description	Ki/IC50	Selectivity/Effects	Refs.
AMS-36	Irreversible	Epoxysuccinyl peptidomimetic (naphthalene methylamine at P3, p-methyl phenylalanine at P2)	<3 nM (reported)	Specific for Cat Z/X carboxypeptidase; reduces microglial neuroinflammation, T-cell migration	[94]
nPrNH-(2S,3S)-tEps-Ile-OH	Irreversible	E-64 derivative ([L-3-trans-(propylcarbamoyl)oxirane-2-carbonyl]-L-isoleucine)	Low μM range	~10-fold over Cat B/L; modest potency	[14]
**Z9** (compound **22**)	Reversible	1-(2,3-dihydrobenzo[b]febs.onlinelibrary.wiley + 1dioxin-6-yl)-2-((4-isopropyl-4H-1,2,4-triazol-3-yl)thio)ethan-1-one	Ki = 2.45 ± 0.05 μM	Selective vs. Cat B/L/H/S; inhibits tumor migration	[100,102]
Compound **1**	Reversible	Triazole-benzodioxine derivative	Ki = 38.4 ± 0.20 μM	Initial screening hit	[100]
Compound **2**	Reversible	Triazole-benzodioxine derivative	Ki = 29.2 ± 2.44 μM	Potent hit; reduces tumor migration	[100]

## Data Availability

Data sharing doesn’t apply to this article as no datasets were created or analyzed during the current study. Data sharing not applicable—no new data generated.

## Abbreviations

The following abbreviations are used in this manuscript:

AD	Alzheimer disease
ALS	Amyotrophic lateral sclerosis
APP	Amyloid precursor protein
AUC	Area under the curve
Cat	Cathepsin
CLIP	Class II-associated invariant chain peptide
DC	Dendritic cell
ECM	Extracellular matrix
ELISA	Enzyme-linked immunosorbent assay
EMT	Epithelial–mesenchymal transition
ERGIC-53	ER-Golgi intermediate compartment protein 53
FAK	Focal adhesion kinase
HCC	Hepatocellular carcinoma
HOG	Human oligodendroglioma cell
HSPC	Hematopoietic stem/progenitor cell
ICAM-1	Intercellular adhesion molecule 1
IFN-γ	Interferon-γ
IL	Interleukin
LAMP1	Lysosome-associated membrane protein 1
LFA-1	Lymphocyte function-associated antigen 1
Mac-1	Integrin αMβ2 (CD11b/CD18)
MFB	Medial forebrain bundle
MHC	Major histocompatibility complex
MS	Multiple sclerosis
NLRP3	NLR family pyrin domain-containing 3
OSA	Obstructive sleep apnea
PAH	Pulmonary arterial hypertension
PBMC	Peripheral blood mononuclear cell
PD	Parkinson disease
PDZ	PDZ domain
PS1	Presenilin 1
PS2	Presenilin 2
RGD	Arg-Gly-Asp
SCMAS	Subunit c mitochondrial ATP synthase
SDF-1	Stromal cell-derived factor 1 (CXCL12)
SNc	Substantia nigra pars compacta
TH	Tyrosine hydroxylase
TLR	Toll-like receptor
TME	Tumor microenvironment
